# The Role of Natural Cytotoxicity Receptors in Various Pathologies: Emphasis on Type I Diabetes

**DOI:** 10.3389/fimmu.2014.00004

**Published:** 2014-01-20

**Authors:** Jonatan Enk, Ofer Mandelboim

**Affiliations:** ^1^The Lautenberg Center for General and Tumor Immunology, Institute for Medical Research Israel-Canada, Hebrew University Hadassah Medical School, Jerusalem, Israel

**Keywords:** NK cells, NCR, NKp46, beta cells, diabetes

## Abstract

Natural killer (NK) cells are innate immune lymphocytes that function mainly as immune sentinels against viral infection and tumorigenesis. NK cell function is governed by inhibitory and activating signals arising from corresponding receptors. A prominent group of activating NK receptors is the natural cytotoxicity receptors (NCRs), which includes NKp30, NKp44, and NKp46. These receptors bind various diverse ligands of pathogenic, tumor, and even self origin. Type 1 diabetes mellitus (T1D) is a multifactorial autoimmune disease, in which insulin-producing beta (β) cells are ablated by the immune system. This killing of β cells is carried out mainly by T cells, but many other immune cells have been implicated in the pathogenesis of this disease. Importantly, NK cells were shown to be key participants in the initial autoimmune attack. It was shown that all β cells from humans and mice, healthy or sick, express an unknown ligand for the activating NKp46 receptor. In this review, we describe the role played by the NCRs in various pathologies with an emphasis on Type I diabetes.

Natural killer (NK) cells are innate immune cells that differentiate, like T- and B-lymphocytes, from the common lymphoid progenitor, in the bone marrow. In 1975, NK cells were described to have an intrinsic capacity to rapidly kill tumor cells (1, 2). Subsequent research revealed that these cytotoxic responses could also be elicited by virally infected non-tumor cells (3). Additionally, NK cells have been recognized to be major producers of Interferon-γ, in both pathological and physiological states. NK cells also produce a variety of other cytokines, both pro-inflammatory (such as tumor necrosis factor-α) and immunosuppressive (such as interleukin-10, IL-10) (4). Indeed, in addition to their role in eliminating pathogen-infected and -transformed cells, NK cells have also been implicated in autoimmune responses, primarily as immune-regulators that can limit or enhance the autoimmune response, both via cytokine signaling and direct interactions with other immune cells. The role of NK cells has been investigated in several different autoimmune conditions including: systemic lupus erythematosus, rheumatoid arthritis, multiple sclerosis, and Type I diabetes (T1D) (5–10).

Natural killer cells, which serve as innate immune sentinels, are found throughout the body in both lymphoid and non-lymphoid tissues. While they reside mainly in the peripheral blood (where they represent 2–18% of the lymphocytes in humans), liver (where NK cells can represent up to 50% of the resident lymphocytes), and spleen, they are also found in the skin, in mucosal tissues such as the lung, in the uterine decidua (11) (where they play a key roles in promoting trophoblast invasion and arterial growth) as well as many other tissues. In addition to their functional role in peripheral non-lymphoid tissues, NK cells can also proliferate and undergo maturational processes in these locations (7, 11–15).

Human NK cells are generally classified into two subgroups based on the expression of CD56 and CD16, which roughly correspond to their activation state. Most circulating NK cells are CD56^dim^CD16^+^ and are considered to be activated. In this state, when NK cells recognize their target cells, they respond by either killing the target or by producing and releasing cytokines. In contrast, CD56^hi^CD16^−^ NK cells, which represent the major population of NK cells found in peripheral lymphoid tissues (16) and in the decidua, respond to stimulation by pro-inflammatory cytokines by producing large amounts of cytokines and acquire cytotoxicity only after prolonged activation. It has been well established that the “immature” CD56^hi^CD16^−^ NK cells differentiate into the more active CD56^dim^CD16^+^ state, which in turn can differentiate and mature further (7, 17–24).

Natural killer cell activity is governed by a delicate balance between activating and inhibitory signals, which arise from corresponding activating and inhibitory receptors. Unlike the antigen-specific, somatically recombined receptors found on T- and B-lymphocytes, NK cell receptors are germline encoded and the activating receptors are of limited repertoire (25).

The inhibitory NK receptors, which predominantly recognize major histocompatibility complex 1 (MHC1, or human leukocyte antigen – HLA) proteins, consist of several receptor subgroups. The largest of these groups is the killer-cell immunoglobulin-like receptor (KIR) in humans, in which two or three immunoglobulin-like domains in the extracellular portion of the receptors recognize specific MHC1 alleles differentially. The KIR genes are organized in a highly polymorphic, multigene family that displays considerable allelic polymorphism and though both activating and inhibitory KIRs exist, the inhibitory alleles are more prevalent (26). Other MHC1 recognizing receptors include the leukocyte immunoglobulin-like receptor subfamily B member 1 (LIR1) and the NKG2A-CD94 receptor complex. Moreover, NK cell tolerance toward normal cells is achieved through the expression of MHC1-binding inhibitory receptors by the NKs. Thus, cells that evade cytotoxic CD8+ T-cell recognition and killing by downmodulating the expression of MHC1, render themselves potential targets for NK cell mediated elimination. In addition, several other inhibitory receptors, which bind to non-MHC1 ligands exist, including CEACAM1, CD300a, and TIGIT (26–31). All the different inhibitory receptors contain one or more intracellular immunoreceptor tyrosine-based inhibitory motifs (ITIMs), which mediate the downstream inhibitory signals by recruiting protein tyrosine phosphatases (32). MHC1 molecules also educate the NK cells to become functionally competent, and NK cells function poorly in the absence of MHC1 (33).

The activating NK cell receptors recognize tumor-, pathogen-, stress-induced, and self-ligands. Unlike the inhibitory receptors, many of the activating NK receptors lack intracellular signaling motifs. Instead, upon binding to their various ligands, these receptors, which belong to various receptor groups, recruit immunoreceptor tyrosine-based activating motif (ITAM)-containing adapters, such as: DAP10, DAP12, FcεRIγ, or CD3ζ, which in turn mediate tyrosine-kinase based downstream signaling (28, 31). The most prominent NK cell activating receptors are the natural cytotoxicity receptor (NCR) family, which includes three Ig-like proteins: NKp30 (34), NKp44 (35), and NKp46 in humans (36, 37). Of these, NKp46 and NKp30 are constitutively expressed by all NK cells, while NKp44 is expressed only after activation. The gene coding for NKp46 is located in the leukocyte-receptor complex on human chromosome 19, while the genes for NKp44 and NKp30 are located on chromosome 6. Upon binding their respective ligands, the NCRs recruit different ITAM-containing adapters to initiate signal transduction: NKp46 can recruit FcεRIγ and CD3ζ, the latter of which is also recruited by NKp30, while NKp44 recruits the adapter DAP12 (28, 38, 39).

Like many of the other activating NK receptors, the NCRs recognize tumor and self-ligands, yet, to-date they are the only NK receptors which have been found to directly recognize pathogen-derived molecules (28). Indeed, the first NCR ligands recognized were the influenza virus hemagglutinin (HA) and the Sendai virus HA-neuraminidase, which are recognized by NKp46 and NKp44 (28, 40). NKp46 also recognizes unknown ligand/s expressed by *Fusobacterium nucleatum* (41) and *Mycobacterium tuberculosis*, NKp44 recognizes the E-protein of Dengue virus and West Nile virus as well as bacterial cell wall components of *Pseudomonas aeruginosa, Nocardia* spp., and *Mycobacteria* spp. (42). NKp30 recognizes the PfEMP-1 protein of *Plasmodium falciparum* (43) and has recently been shown to bind and mediate the killing of various fungal species (44). Poxvirus HA has also been recognized as a target NKp46 and NKp30 (45). Yet, in the case of NKp30, the poxvirus HA serves as an inhibitory ligand, as is also the case with the pp65 protein of HCMV (46). In addition to recognizing pathogen expressed ligands, the NCRs also recognize several other known ligands, including Heparan sulfates, which are recognized differentially by the different NCRs, (47) as well as BAT3 (expressed by stressed cells) and B7-H6 (expressed by tumor cells), which are recognized by NKp30, (48, 49) and MLL5, which has recently been identified as a tumor expressed protein ligand of NKp44 (16). In addition, all the NCRs recognize unknown ligands expressed constitutively by several types of hematopoietic cells (granulocytes, monocytes, and dendritic cells). The reciprocal interaction between these cells and NK cells can result in their mutual activation or, alternatively, this interaction can sometimes lead to NK cell-mediated killing of immature dendritic cells (50–53).

NKp46 is unique amongst the NCRs and is considered to be the most specific NK cell marker. NKp46 is also distinct in that it is the only NCR that has a murine ortholog, named NCR1 (54–56). As such, NKp46 and NCR1 are the most studied of the NCRs. Mice knockouts (KO) for the *NCR1* gene were generated through the insertion of a reporter gene, encoding green fluorescent protein (GFP), into the *Ncr1* locus. While the heterozygous *Ncr1*^+/gfp^ NK cells are haplo-sufficient and display a wildtype (wt) phenotype in the homozygous *Ncr1^gfp/gfp^* mice, *Ncr1* is knocked out and their NK cells lack NCR1 dependent functions (54, 57). Yet, despite this powerful, commercially available tool, the tumor and cellular ligand(s) for NKp46/NCR1 remain unknown.

To address the issue of NKp46 ligand expression, when such ligands are unknown, fusion proteins containing the extracellular portion of NKp46 fused to the Fc portion of human IgG1 have been used for cell and tissue staining. By applying this technique to murine and human tissue samples, it was found that both human and murine insulin-producing beta cells (β cells) constitutively express an NKp46 ligand (57, 58). In addition to β-cells, only two more normal tissues were found to express an NKp46 ligand(s), the salivary glands and hepatic stellate cells (57, 59). However, NKp46 and the other NCRs have also been found to bind to as of yet unknown ligands, of cellular, bacterial, fungal, and viral origin. Although the identities of these ligands remain largely unknown, experimental evidence suggests that each of the NCRs interacts with several distinct ligands (28).

In addition to the NCRs, NK cells express several other activating receptors: CD16 which mediates antibody-dependent cell-mediated cytotoxicity (ADCC); NKG2D which recognizes several stress-induced ligands expressed by cancerous, virally infected, and other stressed cells; as well as several receptors including NKp80, 2B4, DNAM1, NKG2C, and some short tailed KIRs that recognize ligands expressed physiologically on different cell types. In this regard it is important to note that all the activating NK receptors, with the exception of CD16, have been shown to be insufficient on their own in stimulating NK cell cytolytic functions (27, 28, 31).

Thus, NK cell populations, which variably express different activating and inhibitory receptors, may respond differentially upon encountering a potential target cell. However, the underlying principles that control NK cell activation remain the same: activating signals emanating from their corresponding receptors (mediated by tyrosine-kinase based signal transduction pathways), are integrated with repressive signals from inhibitory receptors (mediated by protein phosphatases), culminating in either target cell killing or in unresponsiveness (27, 60).

In the following segments, we will describe the involvement of NK cells in general and NKp46 specifically in T1D to exemplify the complexity of studying the roles of NCRs in human disease. T1D is a multifactorial autoimmune disease in which insulin producing β cells, which reside in the islets of Langerhans in the pancreas, are attacked and killed predominantly by autoreactive T lymphocytes. Indeed, adoptive transfer experiments of T cells from diabetic mice to non-diabetics, confers diabetes via the killing of β cells in the recipient (61). However, other immune cells have also been implicated in diabetes pathogenesis (Figure 1): antibody producing B-lymphocytes have been implicated in maintaining T-cell reactivity toward β cells (62); macrophages, which are recruited to the islets of Langerhans in non-obese diabetic mice (NOD, the most studied animal model for T1D), prior to T-cell arrival, have been shown to be critical for diabetes progression (63); in addition, NK cells have also been implicated in T1D pathogenesis.

**Figure 1:**
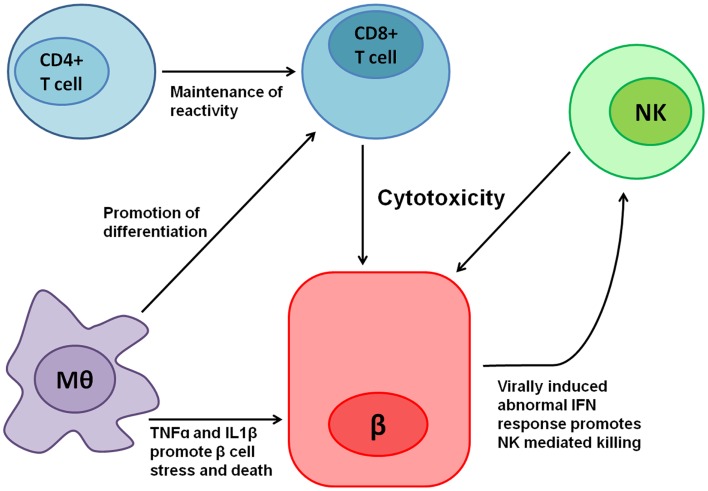
**Various immune cells are involved in T1D pathogenesis**. Cytotoxic CD8+ T cells mediate most of the direct β cell killing, while CD4+ T cells help in maintaining their reactivity. Macrophages (Mθ) release tumor necrosis factor-α (TNFα), IL1β, and other cytokines, which cause β cell stress and promote cell death, in turn contributing to the local inflammation. Moreover, Mθ promote CD8+ T-cell differentiation mediated both by direct interactions and in Trans by cytokines. Local inflammation, β cell stress, and the expression of an unknown ligand for NKp46 contribute to NK cell reactivity toward β cells, which have been shown to kill β cells. In addition, β cells have been shown to exhibit abnormal interferon (IFN) responses when infected by viruses (64), thus potentially contributing even further to NK mediated killing.

The first reports linking NK cells to diabetes came in the 1980s and early 1990s. These reports found that the NK cells from diabetic and diabetic prone rats were more cytotoxic than their counterparts from diabetic resistant rats, from the same genetic backgrounds (65–67). The next reports linking NK cells to diabetes came when experiments demonstrated that antibody-dependent depletion of NK cells (using an anti-asialo GM1 antibody, which depletes NK cells, with “off-target” effects on T cells and basophils (68)) could prevent diabetes development in two separate chemically induced diabetes models: low-dose streptozotocin (LDST) and cyclophosphamide (69, 70). Regardless, in both cases the experiments were conducted in mice, thus widening the scope and validity of the original findings: NK cells were not only capable of killing islet cells, but also seemed to be important in diabetes development in two distinct experimental models. When NOD mice from two genetic backgrounds were studied, one of which develops severe insulitis (inflammation in the islets of Langerhans) that rarely develops into diabetes and the other, which develops insulitis that rapidly progresses to diabetes, it was found that the main difference was a higher proportion of NK cells in the early insulitis immune infiltrates of the diabetes prone mice (71).

As for activating NK receptor presentation in T1D patients, while reduced NKp46 and NKp30 levels were observed only in patients with long-standing disease, a reduction in NKG2D was observed in both newly diagnosed and long-standing patients (72, 73). Moreover, the prevalence of activating KIR genes was higher in T1D patients when compared to HLA-matched controls. As the KIR repertoire expressed by NK cells determines self-tolerance and affects T-cell function, it would indeed be reasonable that the KIR genes influence susceptibility to T1D (73–75). Thus, NK cells were implicated in diabetes development both in animal models and human patients.

The first mechanistic link between NK cells and diabetes was the discovery that β cells from healthy mice, NOD mice, *Psammomys obesus*, and humans all express an NKp46 ligand (57, 58, 76). Although the identity of this ligand still remains unknown, some insights into the biology of the ligand and its interactions with NKp46 and NCR1 have been gained. It was found that two glycosylated amino acid residues located in the extracellular segment of NKp46, Thr^125^ and the Asn^216^, are critical for binding this ligand (58). Importantly, binding of NKp46 to influenza virus HA is dependent on a different glycosylated residue – Thr^225^. With regards to the expression kinetics of the β cell ligand for NKp46, it was shown that in both adult and young, mice and humans, the ligands are expressed constitutively. However, while human β cells display ligand expression even at the earliest embryonic stages in which insulin is expressed, the murine ligand is not expressed by embryonic β cells. Nonetheless, following birth (as early as day 1 post-partum) the NKp46 ligand is expressed by all murine β cells. One possible explanation for the differences in ligand expression between human and murine β cells is that the NKp46 ligand is somehow associated with functional maturation of the β cells, which occurs relatively earlier during human pregnancy (58).

Moreover, the β-cell ligand expression was found to be closely correlated with β cell function. It was found to be present mainly in the insulin granules and to be released to the plasma membrane as β cells degranulate and release insulin. It was also shown that when β cell function starts to falter, as it happens during Type 2 diabetes development, the β cell NKp46 ligand expression is mostly lost, only to return to normal levels if the Type 2 diabetic phenotype is reversed (76).

The expression of the NKp46 ligand is stable in functional β cells and is maintained in the β cells that survive the immune attack in pre-diabetic and diabetic NOD mice. Ligand expression is also maintained during experimental ablation of β cells: it is maintained in the β cells that survived cytotoxic death mediated by diphtheria toxin A and is also seen during subsequent β cell regeneration. Thus, because of the expression of the NKp46 ligand is stable and is seen in all functional β cells examined, β cells are constantly at risk of being attacked by NK cells via NKp46-mediated activation (58, 77).

Indeed, the outcome of β cell recognition by NK cells was exemplified in a series of experiments (58, 59). In agreement with earlier reports from rat NK cell (65), the incubation of human or mouse β cells with their corresponding NK cells caused NK cell degranulation as well as direct killing of the target β cells, as assessed by radioactive killing assays. Importantly, this β cell induced NK degranulation was NKp46 dependent as it was significantly lower in NK cells derived *Ncr1^gfp/gfp^* when compared to NK cells from control *Ncr1*^+^*^/gfp^* mice. Moreover, in agreement with earlier results, human NK cells killed human β cells and this killing was impeded by blocking NKp46 (using anti-NKp46 sera). Thus human and murine β cells are indeed *in vitro* targets for isolated NK cells, and are killed NKp46 dependently (58).

Additionally, NK cells and NKp46 were also implicated in T1D pathogenesis *in vivo*. NK cells isolated from pre-diabetic NOD islet immune infiltrates, stained for CD107a expression, which is a marker for recent NK cell degranulation (78). Moreover, immunization of NOD mice using NKp46-Ig and NCR1-Ig fusion proteins significantly reduced diabetes incidence, without depleting the NK cell population (57). NKp46 involvement in T1D was not specific to NOD mice: when diabetes was induced in WT and *Ncr1^gfp/gfp^* KO mice using a LDST protocol, diabetes development was significantly impaired in mice lacking NCR1. Thus, NK cells and their NKp46 activating receptor facilitate diabetes development *in vivo*, both in induced and genetic models of the disease.

Thus, all functional β cells express a ligand for NKp46 and NK cells kill them, when encountering β cells. However, diabetes does not develop in all individuals because NK cells never encounter β cells under normal conditions [(58) and Figure 2].

**Figure 2:**
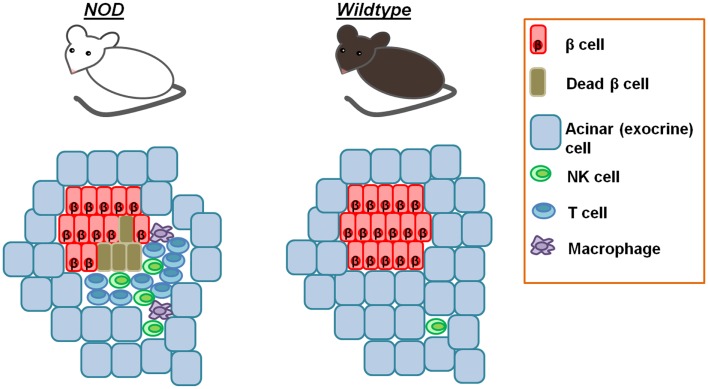
**Pancreatic NK cells in homeostasis and diabetes**. Whereas NK cells are rarely found in the pancreases of normal healthy mice, in the peri-islet immune insulitis infiltrates of NOD mice, NK cells are found starting at the earliest stages of insulitis. This explains why diabetes does not develop in all individuals, despite the intrinsic ability of NK cells to kill β cells.

Natural killer cell involvement in diabetes and the intrinsic NKp46 dependent capacity of NK cells to kill β cells highlight some key aspects of NK cell biology and the roles of the NCRs. Despite powerful tools such as transgenic mice and fusion proteins, many cellular ligands remain unknown, some of which are directly associated with the disease. The identification of the NCR cellular ligands has so far remained very difficult. This is due in part to the fact that each NCR has several different ligands, expressed differentially by different cells and tissues. It remains to be seen whether and how other diseases are influenced by NCRs. Indeed, in the case of liver stellate cells, NK cell recognition of their cognate NKp46 ligand inhibits liver fibrosis (59). Thus, depending on the context the same NCR can serve to exacerbate disease in one setting and ameliorate it in another. Presumably, this has to do with different ligands expression, though this issue remains unclear. Additionally, as the identification of target cells by NK cells is dependent on multiple receptors and different combinations of receptor engagements elicit different responses by the NK cells, the issue of NK cell response to target recognition is vastly complicated.

An early view of NK cells and their receptors identified them as a primordial mechanism of immunity to infectious pathogens and subsequently, the NK cell receptors were speculated to be pattern recognition receptors for pathogens (44, 79). However, the finding that activating NK cell receptors, in general, and the NCRs specifically, bind several distinct ligands of endogenous origin implicates NK cells to be more general immune mediators. It appears that the NCRs are thus general “danger” pattern recognizers that confer complex activities to NK cells.

## Conflict of Interest Statement

The authors declare that the research was conducted in the absence of any commercial or financial relationships that could be construed as a potential conflict of interest.
